# Phylogenomic approaches to detecting and characterizing introgression

**DOI:** 10.1093/genetics/iyab173

**Published:** 2021-11-11

**Authors:** Mark S Hibbins, Matthew W Hahn

**Affiliations:** 1 Department of Biology, Indiana University, Bloomington, IN 47405, USA; 2 Department of Computer Science, Indiana University, Bloomington, IN 47405, USA

**Keywords:** introgression, hybridization, phylogenomics, phylogenomic methods

## Abstract

Phylogenomics has revealed the remarkable frequency with which introgression occurs across the tree of life. These discoveries have been enabled by the rapid growth of methods designed to detect and characterize introgression from whole-genome sequencing data. A large class of phylogenomic methods makes use of data across species to infer and characterize introgression based on expectations from the multispecies coalescent. These methods range from simple tests, such as the *D*-statistic, to model-based approaches for inferring phylogenetic networks. Here, we provide a detailed overview of the various signals that different modes of introgression are expected leave in the genome, and how current methods are designed to detect them. We discuss the strengths and pitfalls of these approaches and identify areas for future development, highlighting the different signals of introgression, and the power of each method to detect them. We conclude with a discussion of current challenges in inferring introgression and how they could potentially be addressed.

## Introduction

The potential for hybridization and subsequent backcrossing between lineages—also known as introgression—has long been understood (Heiser 1949, 1973; Rieseberg and Wendel 1993; Dowling and Secor 1997). Recent hybridization often leads to clear genome-wide patterns in hybrid individuals, allowing for the detection of F_1_, F_2_, and early back-cross hybrids from limited sequence data (Nason and Ellstrand, 1993; Anderson and Thompson 2002). However, many generations of back-crossing can substantially reduce the number of loci retaining a history of hybridization, rendering more ancient hybridization events difficult to detect. As a result, until genome sequencing became widely available, it was often difficult to quantify patterns of introgression effectively and reliably. In part precipitated by the discovery of introgression between archaic human populations (Green *et al.* 2010; Huerta-Sánchez *et al.* 2014), the past decade has seen an explosive increase in the rate of discovery of reticulate evolution across the tree of life (Mallet *et al.* 2016; Taylor and Larson 2019). Although great efforts have been made in recent years to synthesize the biological implications of these discoveries (Ellstrand *et al.* 2013; Hedrick 2013; Harrison and Larson 2014; Racimo *et al.* 2015; Ottenburghs *et al.* 2017; Suarez-Gonzalez *et al.* 2018; Dagilis *et al.* 2021), comparatively little conceptual synthesis has been provided on the accompanying growth in methods used to detect and characterize introgression.

Modern studies of introgression are often predicated on “phylogenomic” datasets. These typically consist of whole-genome or whole-transcriptome sequencing data, collected from or focusing on a single individual in at least three populations or species. Gene trees can be estimated from alignments of individual loci or nonoverlapping genomic windows (neither of which necessarily contain protein-coding genes), resulting in a collection of thousands of tree topologies; most methods also require a species tree to be specified. A common finding from phylogenomic studies is the ubiquity of gene tree discordance—topologies from different loci will disagree with both each other and with the inferred species tree (*e.g.*, Pollard *et al.* 2006; Fontaine *et al.* 2015; Novikova *et al.* 2016; Pease *et al.* 2016; Edelman *et al.* 2019). Although the gene tree topologies from neighboring loci are more likely to be similar (Slatkin and Pollack 2006), discordance occurs even between neighboring loci, as recombination uncouples the history of flanking genomic windows.

When studying introgression, researchers are often interested not just in detection but also the characterization of introgression events. Such characterization can include the direction (identity of donor and recipient populations), the extent across the genome, the timing (in absolute terms or relative to speciation), or the mode (*i.e.*, instantaneous “pulses” of hybridization *vs* continuous gene flow) of introgression. This information is often difficult to glean from a discordant tree at a single locus. When many loci are sampled in a phylogenomic framework, it becomes possible to learn about the general factors causing discordance in a dataset, allowing for introgression to be distinguished from other processes. Data from a rooted triplet of species—or an unrooted quartet—are the minimum requirement to carry out powerful tests for introgression based on gene tree discordance using genome-scale datasets. Importantly, this can be done using only a single haploid sequence per species (here, we use the term “species” loosely to refer to any lineage or population which shows evidence of historical long-term isolation from other such lineages). These approaches range in complexity from summary statistics based on biallelic site patterns or branch lengths to model-based likelihood inference methods.

In this review, we focus on phylogenomic methods for studying introgression, most of which are based on the multispecies coalescent (MSC) model and use data from one sample per species. These methods, despite their simplicity, come with several advantages: (1) gene tree frequencies and branch lengths are fully described under the MSC model using one sample per species, and adding more samples provides little new information with respect to introgression; (2) much of the genealogical signal of introgression detected by these methods is not mimicked by selection (Przeworski *et al.* 1999; Williamson and Orive 2002; Vanderpool *et al.* 2020), making them more robust to non-neutral processes; (3) a description of these methods can help to build biological intuition. For these reasons, modern phylogenomics studies often employ single-sample methods to detect introgression, even when multiple samples per species are available (*e.g.*, Fontaine *et al.* 2015; Pease *et al.* 2016; Edelman *et al.* 2019; reviewed in Dagilis *et al.* 2021).

We provide a detailed conceptual overview of the signals that various introgression scenarios are expected to leave in the genome, and the methods that are designed to detect these signals to infer the presence, timing, direction, and extent of introgression among species. For a more formal statistical treatment of these topics, we direct readers to other reviews: Degnan (2018), Elworth *et al.* (2019), and Jiao *et al.* (2021). We discuss common misuses and misinterpretations of these methods and provide recommendations for best-use practices. Based on these results, we identify areas for future theoretical and methodological advancement, as well as the challenges that remain for interpreting current methods.

## Biological processes that generate gene tree heterogeneity

**Figure 1:**
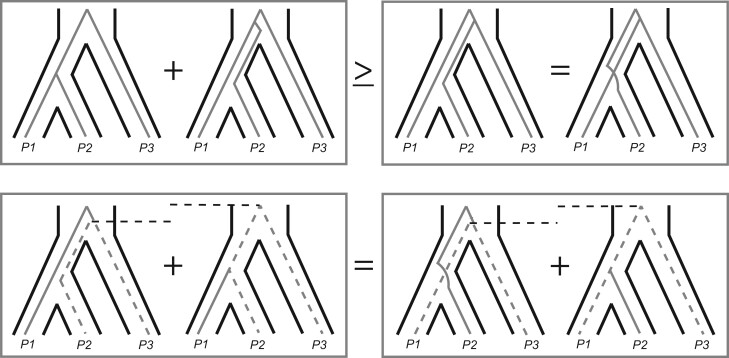
Expected gene tree topologies and coalescence times under ILS only. For a rooted triplet, four topologies are possible (top row): two concordant with the species tree, which can result either from lineage sorting or ILS (top left), and two that are discordant with the species tree and arise from ILS only (top right). The two concordant trees must be at least as frequent as the two discordant trees, which are equally frequent to each other. For nonsister pairs of taxa—either *P2–P3* (bottom left) or *P1–P3* (bottom right)—coalescence is expected to occur at one of two times, depending on whether they coalesce first or second in a gene tree (gray dotted lines). These expected times are symmetrical across gene trees, and so pairwise divergences between the nonsister lineages are expected to be equal when averaged across loci.

We begin our discussion of phylogenomic methods with the simplest possible sampling scheme: genomic data from a single sampled haploid individual from each of three focal species and an outgroup. By “genomic data” we mean data sampled from many loci across the genome, often with the standard assumption of no intra-locus recombination and free interlocus recombination. This data structure will hereafter be referred to as a quartet or rooted triplet. For three ingroup species, *P1*, *P2*, and *P3*, and an outgroup species, *O*, there are three possible tree topologies describing how they can be related: [((*P1*, *P2*),*P3*),*O*], [((*P2*, *P3*),*P1*),*O*], or [((*P1*, *P3*),*P2*),*O*] (Figure  1). In addition to a single bifurcating phylogeny describing the evolutionary history of the quartet, trees can be estimated for each individual locus. The frequencies of each topology across loci are referred to as gene tree frequencies, even when they do not come from protein-coding genes. This heterogeneity in both the topology and branch lengths of gene trees is caused by two different biological processes, incomplete lineage sorting (ILS) and introgression, in addition to errors in gene tree estimation. In this section, we describe the expected effects of ILS and introgression on gene trees in order to explain how tests for introgression work; we discuss the potential impacts of gene tree estimation error in a later section.

### ILS as a null hypothesis for tests of introgression

The phenomenon of ILS, in which two or more lineages fail to coalesce in their most recent ancestral population (looking backwards in time), can result in individual gene trees that are discordant with the species history (Figure  1). Phylogenomic methods must account for this phenomenon to make accurate inferences about introgression. Discordant gene trees occur because, when ILS occurs, it becomes possible for the order of coalescent events to differ from the order of splits in the species phylogeny (Figure  1, top right panel). Gene tree discordance due to ILS is very common in modern phylogenomic datasets (e.g., Pollard *et al.* 2006; Fontaine *et al.* 2015; Novikova *et al.* 2016; Pease *et al.* 2016; Copetti *et al.* 2017; Wu *et al.* 2018a; Edelman *et al.* 2019), though some discordance may be due to gene tree inference errors, especially at older timescales. ILS can arise within phylogenies that contain no introgression events. Because both ILS and introgression can generate many of the same genealogical patterns, it is essential to incorporate ILS into the null hypothesis of tests for introgression.

Fortunately, the effects of the parameters that influence the probability of ILS—time between speciation events and ancestral population size—are well understood from the neutral MSC model (Hudson 1983; Tajima 1983; Pamilo and Nei 1988). For a rooted triplet, the probability that the two sister lineages (*e.g.*, *P1* and *P2* in Figure  1) coalesce in their most recent common ancestral population is given by the formula $1-e^{-\tau }$, where τ is the length of this internal branch in units of 2*N* generations (sometimes referred to as “coalescent units”). Conversely, the probability of ILS (*i.e.*, that they do not coalesce) is $e^{-\tau }$. If ILS occurs, all three lineages (*P1*, *P2*, and *P3*) enter their joint ancestral population. Within this population the coalescent events happen at random, such that lineages leading to each pair of species have a 1/3 chance of coalescing first. This means that the two discordant gene tree topologies are expected to be equal in frequency (Figure  1, top right), with probabilities of ${1/3e}^{-\tau }$ each. In addition, the concordant tree topology can be produced either by lineage sorting with probability $1-e^{-\tau }$ or ILS with probability ${1/3e}^{-\tau }$ (Figure  1, top left). This guarantees that the concordant tree topology will always be at least as frequent as the two discordant trees (Figure  1, top row). These expectations under ILS form the null hypothesis for tests of introgression based on gene tree frequencies.

In addition to gene tree frequencies, ILS affects expected coalescence times, and therefore sequence divergence, between pairs of species. In any population, the expected times to coalescence depends on how many lineages are present (Kingman 1982; Hudson 1983; Tajima 1983). If three lineages are present, the first coalescence is expected to occur 2/3N generations in the past. After this first coalescence—or if only two lineages were present to begin with—the next coalescence is expected a further 2*N* generations in the past. These expectations are equally applicable to current populations as to ancestral populations, but coalescence cannot occur until the lineages under consideration are in a common population. Therefore, expected coalescence times between species always have the time of speciation included as a constant, no matter how far back lineage-splitting occurred (Gillespie and Langley 1979).

For example, the expected time to coalesce between sequences sampled from species *P1* and *P2* in Figure  1 is the time to speciation plus an additional *2N* generations in the past. If this coalescence happens in their most recent common ancestor, the next coalescent event will occur in the common ancestor of all three species, between the *P1/P2* ancestral lineage and the lineage leading to *P3* (Figure  1, bottom row). This event occurs at the time of speciation of *P3* from the *P1/P2* ancestor plus another *2N* generations in the past. If ILS occurs, both coalescent events occur in the common ancestor of all three species, with the first event occurring at the time of speciation plus 2/3*N* generations, and the second event occurring at the time of the first event plus *2N* generations. Note that, if we condition on lineage sorting having occurred, the expected time of the first coalescent event becomes slightly more complicated (see Mendes and Hahn 2018; Hibbins and Hahn 2019 for exact expectations).

The two pairs of nonsister lineages in a rooted triplet (*P1* and *P3* or *P2* and *P3* in Figure  1) can coalesce at one of two times, depending on whether they are the first or second pair to coalesce in a gene tree (there can only be a discordant topology if they are the first to coalesce). Owing to the symmetry of gene tree topology shapes and frequencies, these times are equivalent across loci, leading to the null expectation under ILS that genome-wide divergence between both pairs of nonsister taxa should be equal (Figure  1, bottom row). Finally, each of these coalescence times is expected to follow an exponential distribution around the expected value (Hudson 1983; Tajima 1983). Therefore, coalescence times will be variable, but should still follow the symmetries of the ILS-only model on average.

### The effects of introgression on gene trees

**Figure 2:**
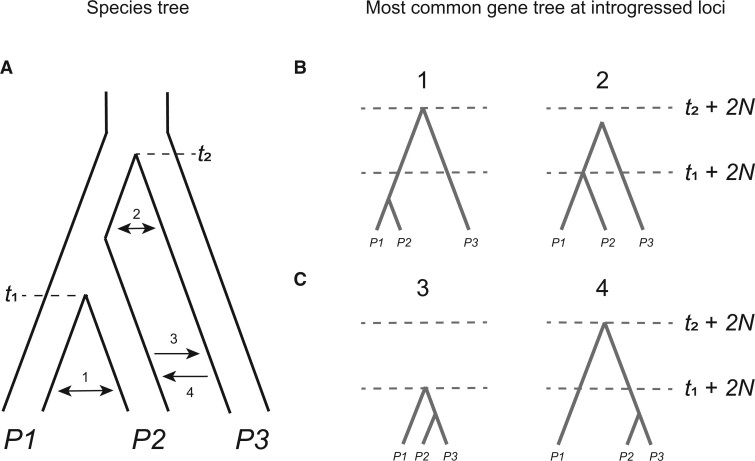
An overview of detectable introgression scenarios for a rooted triplet, and their effects on gene tree frequencies and branch lengths. (A) The species tree relating three lineages, with speciation times *t*_1_ and *t*_2_ labeled. Introgression can occur between extant (1) or ancestral (2) sister lineages, or between nonsister taxa, with *P3* as either the recipient (3) or the donor (4). (B) Gene trees at introgressed loci for introgression between sister lineages. Gray dashes denote the expected coalescence times under ILS only. Introgression between sister taxa reduces divergence between the involved taxa but does not generate discordant gene trees (events 1 and 2). In both trees the expected time to coalescence for pairs of lineages in the absence of introgression is denoted with dashed horizontal lines. (C) Gene trees at introgressed loci for introgression between nonsister lineages. When *P3* is the recipient of introgression (event 3), discordant gene trees are generated uniting *P2* and *P3*. In addition, divergence is reduced between both *P2* and *P3* and between *P1* and *P3*. When *P3* is the donor of introgression (event 4) discordant gene trees are again generated uniting *P2* and *P3*. In this case divergence is reduced only between *P2* and *P3*, while divergence is increased between *P1* and *P2*. In both trees, the expected time to coalescence for pairs of lineages in the absence of introgression is denoted with dashed horizontal lines.

Introgression between two lineages occurs when an initial hybridization event is followed by back-crossing into one or both parental lineages. Hybridization itself—the creation of a hybrid individual—is generally not sufficient to be called introgression, though polyploid or homoploid hybrid species will be identified by many of the same tests described here (*e.g.*, Meng and Kubatko 2009; Blischak *et al.* 2018; Folk *et al.* 2018). Similarly, horizontal gene transfer can also generate discordant gene trees. There are many different introgression scenarios, each with a different effect on the underlying gene trees. While there are well-developed mathematical tools that describe the effects of introgression on gene tree topologies [*e.g.*, the multispecies network coalescent (MSNC); reviewed in Degnan (2018), Elworth *et al.* (2019), and Jiao *et al.* (2021)], we generally do not need the predictions from these models to test for the presence of introgression (with some exceptions discussed below). Instead, because our tests are often simply looking for a rejection of the ILS-only model (see previous section for a description of expected patterns under ILS alone), a general understanding of the key outcomes of introgression will be sufficient. In later sections, we will describe in more detail the specific signatures used to characterize the extent, timing, and/or direction of introgression events. Figure  2 summarizes the scenarios involving introgression that are thought to be most frequent.

As a first key distinction, introgression can occur either between sister lineages (events 1 and 2 in Figure  2A) or nonsister lineages (events 3 and 4 in Figure  2A). Generally, introgression between sister lineages should increase the proportion of concordant gene trees relative to the case of ILS alone. To see why this is, consider introgression event 1 in Figure  2: gene flow after speciation between *P1* and *P2* effectively increases τ, the length of the internal branch separating these two lineages from their common ancestor with *P3*. This is because *P1* and *P2* can now be more closely related at introgressed loci than in the species phylogeny. As discussed in the previous section, the rate of ILS is inversely proportional to the value of τ. Loci with an introgressed history therefore have a reduced probability of ILS because of the increased time for *P1* and *P2* to coalesce. While there are some exceptions to this rule—all of which involve introgression between sister lineages on an internal branch of the species tree (*i.e.*, event 2 in Figure  2; Solís-Lemus *et al.* 2016; Long and Kubatko 2018; Jiao and Yang 2021)—in no cases should gene flow between sister lineages result in one discordant topology becoming more common than others. Because an increase in concordant topologies is also consistent with an ILS-only model with a longer internal branch in the species tree, gene tree frequencies alone cannot tell us whether introgression has occurred between sister lineages.

When introgression occurs between nonsister lineages (events 3 and 45 in Figure  2A), one discordant tree topology can become more common than the other. This asymmetry in discordant tree topologies is one of the clearest signals of introgression. In both events 3 and 4, we expect loci that have introgressed to be more likely to have the gene tree topology [(*P2*, *P3*),*P1*] (Figure  2C). While not all loci following an introgression history will have this discordant topology, the extended period of shared history between *P2* and *P3* makes it more likely for these lineages to coalesce. In general, the strength of the asymmetry in discordant topologies will depend on the net extent, timing, and direction of introgression (Durand *et al.* 2011; Martin *et al.* 2015; Zheng and Janke 2018), as well as the absence of introgression between the other nonsister pair (in which case the other discordant topology would also go up in frequency). Although the same discordant topology will be produced in excess by events 3 and 4 (Figure  2C), note that the resulting branch lengths will differ on average between the two. This difference makes it possible to determine the main direction of introgression between nonsister taxa (see below). Note that while we have drawn gene flow as unidirectional to highlight the fact that this distinction can be made, bidirectional gene flow between these lineages is also biologically plausible.

## Detecting introgression using gene tree frequencies

### The D-statistic

A widely used method for inferring introgression is the *D*-statistic, or—perhaps because there are already so many *D*s in use—what is commonly referred to as the ABBA–BABA test (Green *et al.* 2010). The statistic quantifies biallelic site patterns produced by introgression between nonsister taxa as a proxy for gene tree frequencies. By using site patterns, it avoids the need to infer gene trees from individual blocks of the genome; the test was originally formulated to test for evidence of gene flow between Neanderthals and archaic humans (Green *et al.* 2010; Durand *et al.* 2011), where estimating full gene trees would not have been feasible. Possibly because of this minimal requirement, it is still the most widely used test for introgression (Dagilis *et al.* 2021).

**Figure 3:**
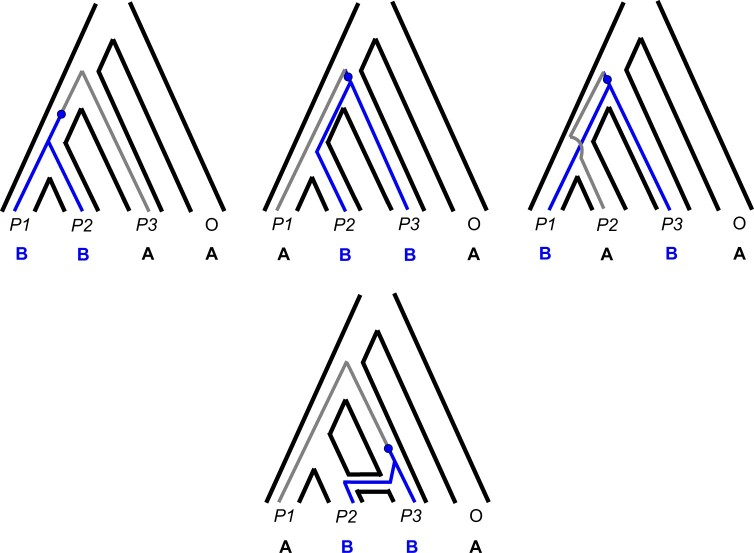
Biallelic site patterns are informative of underlying gene tree topologies. Except for low levels of homoplasy, such patterns can only arise from mutations (blue) on internal branches of the local genealogy. The occurrence of the incongruent site patterns “ABBA” (top middle) and “BABA” (top right) are therefore expected to reflect the frequency of discordant gene tree topologies. With introgression between a specific nonsister species pair, one incongruent pattern (bottom) can increase in frequency over the other due to the underlying asymmetry in gene tree frequencies.

The *D-*statistic counts the occurrence of two configurations of shared derived alleles across three species and an outgroup. Assuming the species tree [((*P1*, *P2*),*P3*)*O*], and denoting the ancestral allele as “A” and the derived allele as “B,” there are two parsimony-informative patterns of discordant sites. The pattern “ABBA” represents sites where *P2* and *P3* share a derived allele, while *P1* and the outgroup have the ancestral allele. The pattern “BABA” represents sites where *P1* and *P3* share a derived allele, to the exclusion of *P2* and the outgroup (Figure  3). For clarity, note that sites supporting the species topology would have the pattern BBAA; however, these are not used in this statistic.

The *D-*statistic assumes an infinite-sites model, meaning that the two discordant site patterns can only arise via single mutations on the internal branches of discordant gene trees (Figure  3, blue dots/branches). Under this assumption, the frequencies of ABBA and BABA site patterns summed across many genomic loci are expected to reflect the frequencies of underlying gene trees. If the number of ABBA and BABA sites differs significantly, then an asymmetry in gene tree topologies is inferred, with introgression occurring between the species sharing the derived state more frequently. Figure  3 depicts the scenario when the site pattern ABBA is more common, implying introgression between *P2* and *P3*.

To make it comparable across studies, the value of the *D-*statistic is typically reported after normalization using the sum of ABBA and BABA pattern counts, giving the following formula:

$$D=\frac{\mathrm{ABBA}-\mathrm{BABA}}{\mathrm{ABBA}+\mathrm{BABA}},$$

where ABBA and BABA represent the number of sites of each type. This statistic has an expected value of *D *=* *0 if there is no gene flow (see “High ILS” simulation condition; Supplementary Figures S2 and S3). When used as a whole-genome test of introgression between nonsister taxa, the *D*-statistic is robust under many different scenarios (Zheng and Janke 2018; Kong and Kubatko 2021), but can be affected by certain forms of ancestral population structure (Slatkin and Pollack 2008; Durand *et al.* 2011; Lohse and Frantz 2014) (see *Distinguishing introgression from ancestral population structure* for more discussion of this issue).

Despite the widespread popularity and relative robustness of *D*, there are several important considerations and limitations to its use, some of which are often overlooked. The first of these concerns how to properly test the null hypothesis that *D *=* *0. The expected site pattern counts of the *D*-statistic can easily be calculated, so it may be tempting to use a parametric test for differences. However, such tests assume that individual observations represent independent samples: this assumption is violated because closely spaced sites often share the same underlying local genealogy, making them nonindependent. The pseudoreplication that results from treating all sites independently leads to inaccurate *P*-values. The solution to this issue is to use a block-bootstrap (or block-jackknife) approach to estimate the sample variance and then to calculate the *P*-value (Green *et al.* 2010). This approach correctly accounts for correlations within blocks of adjacent sites.

Although formulated as a genome-wide test, there are cases where the *D*-statistic has been applied to look for introgression in smaller genomic windows (*e.g.*, Kronforst *et al.* 2013; Zhang *et al.* 2016; Wu *et al.* 2018b; Grau-Bove *et al.* 2020). However, the genome-wide expectation under ILS alone that *D *=* *0 does not hold true for smaller genomic windows. Since a single nonrecombining locus contains a single genealogy by definition, it is only capable of generating one parsimony-informative biallelic site pattern (again assuming an infinite-sites mutation model). The consequence is that the value of *D* at a single locus can only be +1, 0, or −1, depending on the local genealogy (*i.e.*, only ABBA, BBAA, or BABA). Therefore, even in ILS-only scenarios, there will be regions of the genome with extreme values of *D*, either positive or negative. This situation is more likely to occur in regions of low recombination, as in these regions even large genomic windows may only contain a small number of independent genealogies. Highlighting this problem, Martin *et al.* (2015) found that the variance of *D* is inflated in regions of low recombination, resulting in an excess of false positives if tests were to be performed on a per-window basis. Similar caution is warranted when applying *D* to inversions, as the entire inversion can act as a single locus (*cf*., Fuller *et al.* 2018). For these reasons, while it may be informative to plot the value of the *D-*statistic along chromosomes, tests using *D* should be applied only to whole genomes, or at least to genomic regions that are sufficiently large to guarantee sampling a large number of underlying genealogies.

The *D*-statistic does not provide any information about introgression other than its presence or absence. While its value does increase with the proportion of introgressed loci, it is not a good estimator of this quantity, tending to greatly overestimate the true value (Martin *et al.* 2015; Pfeifer and Kapan 2019; Hamlin *et al.* 2020). In addition, the sign of *D* is sometimes interpreted as providing information on the direction of introgression, though it can only identify which taxa are involved, and not the donor and recipient populations. For example, a significant *D-*statistic implying introgression between *P1* and *P3* could involve the *P3* → *P1* direction, the *P1 → P3* direction, or some combination of the two. *D* has more power to detect introgression in the *P3* → *P1* direction (see simulation conditions “P1 into P3” and “P3 into P1”; Supplementary Figures S2 and S3), but can detect it in either direction. Lastly, the *D-*statistic is agnostic to the timing of introgression (as long as it is postspeciation) and may yield a positive result under a variety of scenarios, including instantaneous “pulses” of introgression (*i.e.*, the MSNC model), hybrid speciation/admixed population formation, or gene flow over continuous periods of time (*i.e.*, the isolation-with-migration, or “IM” model; Wakeley and Hey 1998; Nielsen and Wakeley 2001).

Overall, the *D*-statistic is a very reliable genome-wide test for introgression, but alternative methods are needed to characterize any detected introgression events in more detail.

### Inferring the extent and direction of introgression using derived allele counts

Many researchers are interested not only in the presence or absence of introgression, but in quantifying its magnitude across the genome and in identifying the donor and recipient populations. Here, we use “extent” to refer to the proportion of the genome that originates from a history of introgression. This is also sometimes referred to as the “inheritance probability” or “admixture proportion.” Alternatively, in the IM framework, the movement of migrant individuals over continuous time is characterized by a “rate” of introgression (Wakeley and Hey 1998; Nielsen and Wakeley 2001).

Accurate estimates of the extent and direction can be obtained by considering additional biallelic site patterns to ABBA and BABA. Many such methods exist, and discussing them at length is unnecessary for the scope of our review; here we simply mention a few of these approaches and direct readers to the relevant literature. As mentioned earlier, simply using the *D-*statistic does not provide an unbiased estimation of the extent of introgression (Martin *et al.* 2015; Pfeifer and Kapan 2019; Hamlin *et al.* 2020). A recently proposed extension of *D* called *D*_p_ (Hamlin *et al.* 2020) adds the counts of BBAA sites to the denominator to form:

$$D_{p}=|\frac{\mathrm{ABBA}-\mathrm{BABA}}{\mathrm{BBAA}+\mathrm{ABBA}+\mathrm{BABA}}|.$$

Taking the degree of asymmetry as a fraction of the total number of parsimony-informative biallelic sites brings *D*_p_ conceptually closer to estimating a genome-wide introgression proportion (see the *D*_f_ statistic of Pfeifer and Kapan 2019 for a similar approach).

Another common approach is to compare the observed value of an introgression test statistic to the value that would be expected under a scenario where the entire genome was introgressed. This expected value can be obtained by estimating the statistic using the same species, or even the same sample, in both the donor and recipient positions. The *F*_4_-ratio or α (Green *et al.* 2010; Patterson *et al.* 2012; Peter 2016) and *f*_d_ (Martin *et al.* 2015) statistics take this approach. The α statistic requires data from five samples and assumes an admixed population with two parent populations, while *f*_d_ assumes complete homogenization of allele frequencies under total introgression, making it applicable to a quartet. *HyDe* (Blischak *et al.* 2018; Kubatko and Chifman 2019) estimates the extent in a similar way under a hybrid speciation scenario using linear combinations of derived site patterns. The assumptions of *F*_4_ and *HyDe* are somewhat restrictive and are not likely to be reflective of the majority of introgression in nature (Schumer *et al.* 2014). However, *HyDe* gives highly accurate estimates of the extent of introgression when its assumptions about hybridization are met, and still provides reasonable estimates for the extent when these assumptions are violated (Kong and Kubatko 2021).

Unless additional assumptions are made, there is not enough information contained in the frequency of gene tree topologies (*i.e.*, site pattern counts) alone to estimate the direction of introgression in a quartet or rooted triplet. However, if a sample is obtained from a fifth species (Eaton and Ree 2013; Pease and Hahn 2015) or if multiple samples per species are available for the quartet (Martin and Amos 2021), then it is possible to infer the direction of introgression. The “partitioned *D*-statistics” of Eaton and Ree (2013) were the first attempt to infer the direction of introgression in a five-taxon phylogeny. Unfortunately, redundant site pattern counts make the results of this directionality test uninterpretable. The *D*_FOIL_ method of Pease and Hahn (2015) resolves this problem by setting up a system of four *D*-statistics, explicitly testing each of the 16 possible introgression events and directions. *D*_FOIL_ assumes that the five-taxon phylogeny is symmetric, with two pairs of sister species. In this particular configuration of species it becomes possible to polarize introgression events because the direction of introgression affects relationships between the donor and both the recipient species and its sister taxon. Unfortunately, *D*_FOIL_ does not work if the species tree is an asymmetric, or “caterpillar,” tree.

### Inferring introgression events from estimated gene trees

While methods based on site patterns can be powerful, there are also fundamental limitations to the kinds of data they can be applied to. First, as mentioned earlier, a key assumption of the *D-*statistic is an infinite-sites model of mutation. When applied to closely related, extant species, this assumption is likely to hold. However, with increasing divergence times it becomes more likely that ABBA and BABA site patterns can accumulate due to convergent substitutions, and thus will no longer reflect underlying gene tree topologies. This can potentially lead to false positives if there is variation in substitution rates among samples. For this reason, site patterns may not be a reliable way to test for introgression between more distantly related extant species, or along branches deeper in a species tree. Second, as the number of sampled species increases, the number of possible trees and quartets increases super-exponentially (Felsenstein 2004). This makes it impractical to apply quartet-based methods to trees with many taxa.

A solution to these problems is to estimate gene tree topologies directly. While substitution rate variation can still lead to systematic errors in gene tree inference, this approach should be more robust than simply using site patterns because explicit tree inference methods such as maximum likelihood can better accommodate convergence on long branches (Swofford *et al*. 2001). Once gene trees have been estimated from many loci, the counts of discordant topologies can be used in much the same way as ABBA and BABA sites are in the *D* test. In fact, Huson *et al.* (2005) proposed such a test comparing alternate tree topologies in a triplet, using a statistic they called Δ. Significance in genome-scale datasets can be evaluated by bootstrap-sampling the estimated gene trees (Vanderpool *et al.* 2020) or by assuming a χ^2^ distribution (Suvorov *et al.* 2021), with Δ=0 again representing the null hypothesis under ILS alone. While Δ still has the potential to be affected by sources of technical error such as systematic bias in gene tree inference—and may have limited power to detect very ancient introgression—it has the advantage of being more robust to the infinite-sites assumption and allows for testing of introgression along deep, internal branches of a phylogeny, while maintaining power comparable to *D* for more recent introgression scenarios (Supplementary Figure S3). Therefore, Δ represents a straightforward way to test for introgression using a small number of additional assumptions.

Estimated gene trees can also be used as input to phylogenetic network methods. These methods construct a likelihood or pseudolikelihood function that is explicitly derived from a phylogenetic network model, for which parameters can then be estimated using either maximum likelihood or Bayesian approaches. The program *PhyloNet* has methods that infer networks directly from gene tree topologies using either maximum likelihood (*InferNetwork_ML*; Yu *et al.* 2014) or maximum pseudolikelihood (*InferNetwork_MPL*; Yu and Nakhleh 2015). Similarly, *SNaQ* (Solís-Lemus and Ané 2016) estimates a network with reticulation edges *via* maximum pseudolikelihood using quartet concordance factors (Baum 2007)—essentially just the counts of the three possible unrooted tree topologies. We will discuss phylogenetic network methods in more detail in *Likelihood methods for detecting introgression.*

## Detecting introgression using coalescence times

While much can be learned about introgression from the frequency of gene tree topologies alone, including additional information about the distribution of coalescence times can lead to much richer inferences. Some advantages of including coalescence times include more flexibility in inferring introgression between nonsister species, detection of introgression between sister taxa, and distinguishing introgression from ancestral population structure. In the following sections, we expand on the expected effects of introgression on coalescence times and branch lengths, followed by a description of how this information is used in concert with gene tree frequencies to make inferences about introgression.

### Detecting introgression using signals of pairwise divergence

Just as was the case for gene tree topologies, it is possible to make inferences about introgression by studying violations of expected patterns of pairwise coalescence times under an ILS-only model. As previously mentioned, one of these expected patterns is a symmetry in coalescence times between the two pairs of nonsister taxa in a quartet (Figure  1, bottom). If one pair of nonsister taxa has more recent coalescence times on average than the other, postspeciation introgression between that pair is a likely explanation. Coalescence times can be approximated using simple measures of pairwise sequence divergence, assuming an infinite-sites model (or at least that genetic distance is proportional to coalescence time). Therefore, one of the simplest ways to test for introgression is to test for an asymmetry in pairwise sequence divergence. This logic has been informally applied to test for introgression (Brandvain *et al.* 2014) and has recently been formalized in several test statistics including *D*_3_ (Hahn and Hibbins 2019) and the branch-length test (Suvorov *et al.* 2021). *D*_3_ is straightforward and has the following definition (changed from the original to be consistent with the notation used here):

$$D_{3}\;=\;\frac{d_{P2P3}\;–\;d_{P1P3}}{d_{P2P3}\;+\;d_{P1P3}},$$

where *d* denotes the genetic distance between the specified populations. This statistic takes the same general form as the *D*-statistic, where the relevant difference in the numerator is normalized by the sum of the two values in the denominator. Like the *D-*statistic, significance of *D*_3_ can be evaluated using a block-bootstrap. A major advantage of *D*_3_ over site-pattern-based tests is that it does not require data from an outgroup—it only needs one haploid sequence from three ingroup species. As with *D*, *D*_3_ can only detect introgression between nonsister lineages and has comparable power under this scenario (Supplementary Figure S3).

### Characterizing introgression using estimated gene trees with branch lengths

Using pairwise divergences between only nonsister taxa ignores information about the full distribution of coalescence times within different gene tree topologies. More information is contained within these branch lengths, allowing for estimation of the timing and direction of introgression in a quartet. As with pairwise measures, we assume that branch lengths from gene trees are a good proxy for coalescence times. However, branch lengths can be affected by other factors, such as substitution rate variation, selection, sequencing error, and/or gene tree estimation error. Care must therefore be taken when applying all methods that use this information, including the likelihood methods described later. Despite these caveats, several signals appear to be robust to many perturbing factors.

**Figure 4:**
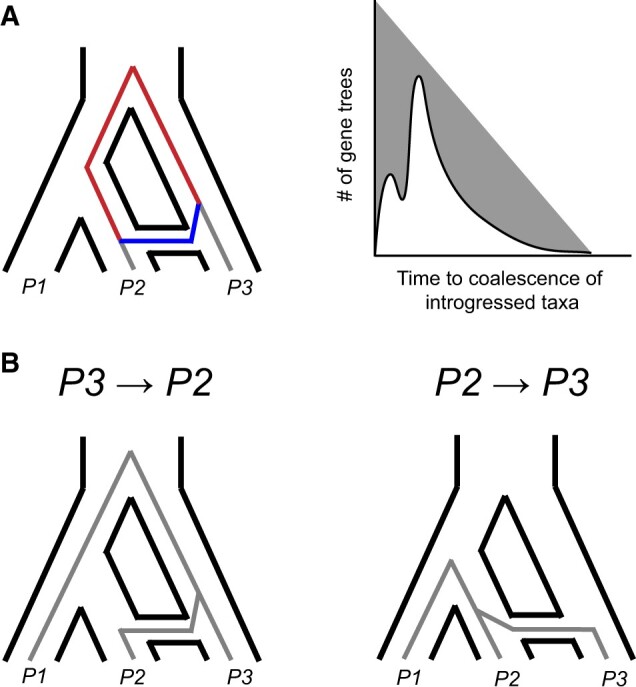
Coalescence times provide information on the timing, direction, and presence of introgression. (A) Postspeciation introgression between *P2* and *P3* allows them to coalesce more quickly at introgressed loci (blue). This reduces their whole-genome divergence relative to *P1* and *P3*, an asymmetry that can be used to test for introgression. Since coalescence can now occur at one of two times, after introgression (blue) or after speciation (red), it also results in a bimodal distribution of coalescence times across loci (right figure). The more recent peak of this distribution can be used to estimate the timing of introgression. (B) The direction of introgression between *P2* and *P3* affects the time to coalesce of *P1* and *P3* at introgressed loci. *P2* → *P3* introgression allows *P1* and *P3* to coalesce more quickly (right), reducing their divergence at introgressed loci.

Because introgressing taxa can coalesce via either introgression (Figure  4a, blue) or speciation (Figure  4A, red) depending on the history at a locus, a bimodal distribution arises when coalescence times are measured across loci (Figure  4A). This distribution is not expected under ILS alone and can therefore be used to test for introgression. In addition, the more recent peak provides information about the timing of introgression, while the frequency of gene trees under this peak compared with the older peak provides information on the extent of introgression. This approach to characterizing introgression is implemented in the software *QuIBL* (Quantifying Introgression via Branch Lengths; Edelman *et al.* 2019).

The direction of introgression uniquely affects the coalescence times of the nonsister pair of species uninvolved in introgression (Figures  2C and 4B). For example, the direction of introgression between *P2* and *P3* has predictable effects on the coalescence time between *P1* and *P3*. When introgression occurs from *P3* into *P2* (Figure  4B, left), *P2* traces its ancestry through the *P3* lineage at introgressed loci (note that while the direction of introgression is typically described forward in time, the coalescent process occurs backwards in time). Because of this, divergence between *P1* and *P3* is unchanged by introgression in this direction. In contrast, when introgression is from *P2* into *P3* (Figure  4B, right), *P3* traces its ancestry through the *P2* lineage at introgressed loci. This allows *P3* to coalesce with *P1* earlier than it normally would, which decreases the divergence between *P1* and *P3*.

These genealogical processes lead to general predictions that can be used to infer the primary direction of introgression between taxa. Gene trees that are concordant with the species tree can be used as a baseline for the expected amount of *P1–P3* divergence; although these trees can arise from ILS at introgressed loci, the effect of the direction will not be manifest since they are concordant. By comparing this baseline divergence to the amount of *P1–P3* divergence in gene trees consistent with a history of introgression, the direction of introgression can be inferred. Lower *P1–P3* divergence in the latter class of trees provides evidence for *P2* → *P3* introgression, but does not necessarily rule out the other direction (*i.e.*, there could simply be less gene flow in the other direction). Alternatively, if *P1–P3* divergence is the same in both topologies, then introgression is primarily *P3* → *P2*. This logic to polarizing introgression is used by the *D*_2_ statistic (Hibbins and Hahn 2019) and the *DIP* method (Forsythe *et al.* 2020).

Finally, *PhyloNet’*s *InferNetwork_ML* method (Yu *et al.* 2014) is able to infer phylogenetic networks with reticulation edges (*i.e.*, discrete introgression events) from gene trees with branch lengths using maximum likelihood. See *Likelihood methods for detecting introgression* for a more detailed discussion.

### Distinguishing introgression from ancestral population structure

Asymmetric gene tree topology frequencies can arise from certain kinds of ancestral population structure (Slatkin and Pollack 2008; Durand *et al.* 2011; Lohse and Frantz 2014). The scenario that generates asymmetries imagines that the population ancestral to all three species is split into at least two subpopulations, such that the ancestors of *P3* are more closely related to either the ancestors of *P1* or *P2* (but not both) (Supplementary Figure S1A). Because the gene tree topologies in this ancestral species will be skewed toward relationships joining *P3* and one of the sister lineages, this scenario can lead to a significant asymmetry in gene tree topologies even in the absence of postspeciation introgression (Durand *et al.* 2011). This will also result in a slight asymmetry of genome-wide pairwise divergence times, since the more common discordant tree will contribute more to the average value. All of this means that ancestral structure can result in false positives when testing for introgression using simple patterns of asymmetry.

Fortunately, while these two scenarios are indistinguishable using only gene tree topologies alone, they are distinguishable when using the distribution of branch lengths. Under ancestral population structure, divergence between the sister taxa in whichever discordant gene tree becomes more frequent will be higher than it would be under introgression. Lohse and Frantz (2014) incorporated the expected branch length differences in these two models into a maximum likelihood framework, which was then used to confirm the signal of human–Neanderthal introgression that was originally uncovered by the *D*-statistic. Additionally, ancestral population structure is not expected to result in a bimodal distribution of coalescence times. This means that methods capable of detecting two peaks of coalescence, such as *QuIBL* and *PhyloNet*-based methods that use trees with branch lengths or sequence data directly (and possibly other likelihood methods), should also be robust to the effects of population structure.

### Detecting introgression between sister species

Introgression between sister species is very difficult to detect using a single haploid sequence from each species. The classic asymmetry patterns described in previous sections do not apply in this scenario, either for gene tree topologies or coalescence times. While introgression between sister species should lead to an increased variance in coalescence times compared with an ILS-only model, this signal is easily confounded by other processes such as nonequilibrium demography or linked selection (Cruickshank and Hahn 2014; Roux *et al.* 2016; Sethuraman *et al.* 2019). These limitations have typically been addressed by combining two alternative sources of information: (1) multiple sequences for each of the two introgressing species and (2) local reductions in between-species divergence relative to a genome-wide baseline.

Most available methods for inferring introgression between sister taxa are not phylogenomic in multiple senses: they typically require polymorphism data, they often identify locally introgressed regions rather than genome-wide signals, and they do not explicitly test against an ILS-only case. Genome scans using summary statistics such as *F*_ST_ (Wright 1931) and *d*_xy_ (Nei and Li 1979) are common, though relative measures of divergence such as *F*_ST_ are confounded by natural selection when used for this task (Charlesworth 1998; Noor and Bennett 2009; Nachman and Payseur 2012; Cruickshank and Hahn 2014). There are multiple statistics based on minimum pairwise distances between multiple haplotypes in two species that avoid problems caused by selection (Joly *et al.* 2009; Geneva *et al.* 2015; Rosenzweig *et al.* 2016), and new machine learning methods combine multiple summary statistics into a single comparative framework that is powerful and robust (*e.g.*, Schrider *et al.* 2018). However, these methods also usually require coalescent simulation under known demographic history to evaluate patterns of introgression, and this information is not always available.

None of the aforementioned limitations mean that genome-wide tests with one sample per species are not possible. Introgression between sister taxa—at least when it occurs in relatively discrete pulses—should result in the same multimodal distribution of coalescence times described above for nonsister taxa. This may be the most promising avenue for a genome-wide test of sister introgression when only one sample per species is available, since coalescence times for two species should follow an exponential distribution under ILS alone. Nevertheless, no methods have been developed to date that explicitly test for this pattern (*QuIBL* can only infer it for nonsister taxa). However, *PhyloNet’*s *InferNetwork_ML* method appears to be capable of reliably inferring introgression (including estimating the timing and extent) between sister taxa using gene trees with branch lengths using this signal (Yu *et al.* 2014) (Supplementary Figures S3 and S5) at least when nested within a tree containing more taxa. Regardless, multiple sequences per locus for each species may be necessary to infer the direction of introgression between sister taxa.

## Likelihood methods for detecting introgression

Perhaps the most powerful phylogenomic methods for inferring introgression are those that use model-based maximum likelihood or Bayesian inference. These methods can be constructed from a variety of different introgression models, can estimate a variety of different parameters, and can be applied to different types of data. Some methods infer introgression directly from a multiple sequence alignment, while others use estimated gene trees; some are based on the MSNC framework for modeling introgression, while others use the IM model; finally, some perform full likelihood calculations, while others estimate approximate likelihoods or pseudolikelihoods. Common to all these approaches is the ability to widely search the space of possible introgression scenarios, making the best possible use (in principle) of available datasets to estimate a phylogenetic network.

Likelihood methods for inferring introgression generally use one of two underlying models: either the MSNC model (Meng and Kubatko 2009) or the IM model (Wakeley and Hey 1998; Nielsen and Wakeley 2001). The models are quite similar, differing mainly as to whether introgression occurs in discrete pulses (MSNC) or over a continuous time interval (IM). The models provide expectations for the probability and coalescence times of gene tree topologies under ILS and introgression. These expectations—sometimes combined with models for sequence evolution along trees—allow maximum likelihood or Bayesian inference to be applied to either an inferred set of gene trees or to a set of sequence alignments. From these data, methods can infer the taxa involved in introgression, as well as the extent, timing, and direction of introgression.

Methods that use more data can provide more information, though this comes at a computational cost. Two methods implemented in *PhyloNet*, *InferNetwork_ML* (Yu *et al.* 2014) and *MCMC_GT* (Wen *et al.* 2016), can use gene trees without branch lengths, while *InferNetwork_ML* can also use trees with branch lengths. If branch lengths are not provided, only introgression between nonsister lineages can be identified (as with summary statistics such as *D*), with accurate estimates of the extent and potentially the direction of introgression. With branch lengths, the timing of introgression can also be accurately estimated, along with the identification of introgression between sister lineages. Using full sequences from each locus rather than gene trees can provide still more information, although maximum likelihood inference is only possible in the simplest scenarios (*e.g.*, Lohse and Frantz 2014; Dalquen *et al.* 2017). Instead, most methods that take sequence data as input use Bayesian approaches for inference. These methods include the MSNC-based *MCMC_SEQ* (Wen and Nakhleh 2018) and *MCMC_BiMarkers* (Zhu *et al.* 2018) methods in *PhyloNet*, the *SpeciesNetwork* (Zhang *et al.* 2018) method in *BEAST2*, and the *MSci* method in *BPP* (Flouri *et al.* 2020). Examples of IM-based Bayesian methods include *IMa3* (Hey *et al.* 2018) and *G-PhoCS* (Gronau *et al.* 2011). While all of these methods can in principle use multiple samples per species, this provides limited additional statistical power; as we discuss next, using multiple samples also comes at a significant computational cost.

A major disadvantage of maximum likelihood and Bayesian methods for full inference of phylogenetic networks is their computational performance on larger datasets. For example, the *InferNetwork_ML* method can only be practically applied to datasets of up to 10 species (Hejase and Liu 2016). Bayesian approaches to inferring networks scale especially poorly and are limited to datasets of dozens to hundreds of loci (Wen and Nakhleh 2018; Zhang *et al.* 2018; Flouri *et al.* 2020). Some methods have addressed this problem by estimating approximate likelihoods or pseudolikelihoods. The *InferNetwork_MPL* (Yu and Nakhleh 2015) method in *PhyloNet* and *SNaQ* (Solís-Lemus and Ané 2016) both maximize the pseudolikelihood of a set of gene tree topologies. By using pseudolikelihoods, these methods can be applied to larger datasets with more than ten species and thousands of loci (Hejase and Liu 2016; Solís-Lemus and Ané 2016). However, in some regions of parameter space the phylogenetic network is unidentifiable with these methods; that is, many different combinations of network parameters could be equally consistent with the observed data. These pseudolikelihood methods are also not ideal for use with information criteria, which makes it challenging to evaluate the fit of different inferred networks (see *Inferring the number of introgression events*). Finally, some performance can be gained in Bayesian approaches by fixing parameters of the phylogenetic network to reduce the space of possible solutions (*e.g.*, fixing the network topology; Flouri *et al.* 2020).

The richness of parameters estimated by likelihood methods can also be a double-edged sword, as these inferences are only possible with relatively strong assumptions. In addition to assuming no recombination within loci and free recombination between loci, all methods assume that sequences are evolving neutrally. While many methods make assumptions about neutrality, those that detect introgression using only gene tree topologies are quite robust to this assumption (Przeworski *et al.* 1999; Williamson and Orive 2002; Vanderpool *et al.* 2020). In contrast, the effect of various forms of selection is to cause changes in the distribution of gene tree branch lengths (Adams *et al.* 2018), a change that can be interpreted as introgression by full likelihood methods. This is especially true for inferences of introgression between sister lineages, where information on gene tree topologies is often not useful in distinguishing between these two scenarios (Ewing and Jensen 2016; Roux *et al.* 2016). Since interpreting likelihood methods can be difficult under such circumstances, we recommend complementing these analyses with other approaches that are formulated to be more robust to common model violations. Despite these limitations, likelihood methods for inferring introgression can have many advantages in terms of the power and richness of inference when compared with simpler approaches.

## Challenges for inferring introgression

### Dealing with phylogenetic uncertainty in introgression analyses

Most methods for inferring introgression require that the species phylogeny is known or can be inferred accurately. More precisely, they require a model of the possible histories of coalescence of samples in the absence of introgression, against which introgression hypotheses can be tested. However, for both technical and biological reasons, a single phylogeny often cannot be inferred accurately and/or with a high confidence. If the wrong species tree is chosen, then introgression may be erroneously inferred. In the case where certain regions of the phylogeny are poorly resolved, one approach is to permute only the poorly resolved regions in different introgression analyses, leaving the more confidently resolved “backbone” constant (Beckman *et al.* 2018; Pease 2018). Alternatively, it may be that the wrong species phylogeny is inferred with high confidence; in this case, careful examination of local genealogical patterns and coalescence times can uncover which histories correspond to speciation *vs* introgression (Fontaine *et al.* 2015; Forsythe *et al.* 2020). Finally, likelihood methods should be less vulnerable to uncertainty since the phylogeny and introgression events are typically coestimated. However, computational and visual representations of these results can often be uninformative or misleading with regard to the true species branching order (see *Distinguishing among models of introgression*).

### Evaluating introgression from unsampled ghost lineages

**Figure 5:**
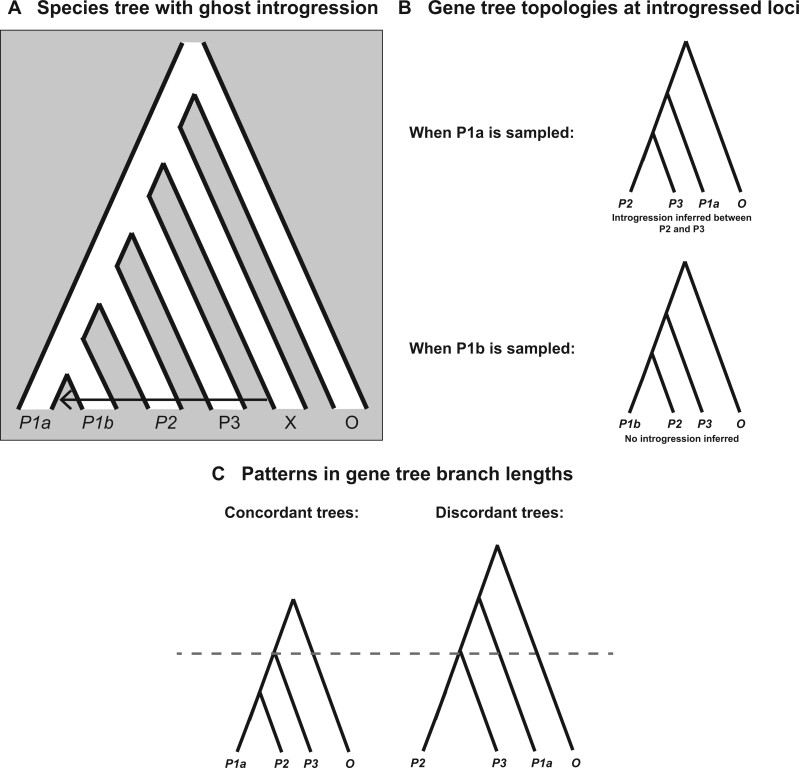
Understanding and detecting ghost introgression. (A) A scenario of ghost introgression from an unsampled outgroup lineage, *X*, into *P1a*. (B) When ghost introgression has occurred and a quartet including *P1a* is sampled, introgression may be erroneously inferred between *P2* and *P3.* This occurs because at some introgressed loci *P1a* will be more distantly related to both *P2* and *P3*, leading to an excess of discordant trees with *P2* and *P3* sister to one another (top). If instead a quartet including *P1b* is sampled, there should no longer be an excess of discordant trees (bottom). (C) Ghost introgression should also be detectable via a change (or a lack of change) in branch lengths. True introgression between *P2* and *P3* should cause them to be more similar; *i.e.*, shorter branch lengths separating them in discordant trees. In contrast, ghost introgression will not make them more closely related in discordant trees than in concordant trees on average. Similarly, the distance between *P1a* and all ingroup lineages will be higher when it is the recipient of ghost introgression from an outgroup.

In studies of introgression, there is always the possibility that the species being studied may have exchanged genes with unsampled “ghost” lineages. These lineages may be unsampled because appropriate specimens were not available for sequencing, because they are currently extinct, or simply because they are unknown taxa. Regardless of their origin, introgression from a distant ghost lineage into a sampled lineage can generate gene tree asymmetry in a rooted triplet. In the scenario considered here (Figure  5A), the ghost lineage is the donor of introgressed alleles into species *P1a*. As a result, at some introgressed loci *P2* and *P3* will appear to be sister lineages (Figure  5B), possibly resulting in an inference of introgression.

Our simulation study (Supplementary Figures S2 and S3), in addition to recent work from Tricou *et al.* (2021), demonstrates that introgression between a ghost lineage and a sampled taxon can result in significant tests for introgression, using both summary statistic and likelihood approaches. While introgression has indeed occurred, the problem is that the timing, direction, and identity of lineages involved in introgression may all be inferred incorrectly. As with results from sampled taxa, significant results are most likely to occur when the ghost taxon is not sister to the species it is exchanging genes with and when the ghost taxon is the donor of introgressed alleles rather than the recipient (Supplementary Figure S3).

There are several approaches researchers can take to detect the presence of ghost introgression. If multiple ingroup lineages are available for testing—but only one of them has been the recipient of introgression—switching the species used in the quartet being tested can reveal ghost introgression. Imagine we have two lineages available to serve as species *P1*: *P1a* and *P1b* (Figure  5A). These lineages should ideally be different species, or at least divergent populations of the same species, that have the same placement in the quartet. *P1a* is the recipient of introgression from an unsampled lineage, *X*, which is more distant than *P3*. If species *P1a* is sampled, we may incorrectly infer introgression between *P2* and *P3* (Figure  5B). In contrast, *P1b* is uninvolved in ghost introgression; if the quartet [((*P1b*, *P2*),*P3*),*O*] is tested for introgression, the result should no longer be significant (Figure  5B). Such a result would be consistent with ghost introgression into *P1a*. If both quartets are significant, this would rule out ghost introgression into *P1a* alone, but could still be explained by ghost introgression into the ancestor of *P1a* and *P1b*.

Given an excess of gene trees with *P2* and *P3* sister to one another, another sign of ghost introgression is that the genetic distance between *P2* and *P3* at discordant loci will not be reduced relative to concordant loci, as would occur if they were truly exchanging alleles (Figure  5C). Although the *D*_3_ statistic is still significant under ghost introgression (Supplementary Figure S3), this is because *P3* is also being compared with *P1*. A simple comparison of the distance between *P2* and *P3* at concordant and discordant loci should reveal if there is any signal of ghost introgression. Conversely, the presence of exceptionally divergent haplotypes in *P1* that are unlikely to have originated from known extant species are also consistent with ghost introgression (Figure  5C). In fact, most known cases of putative ghost introgression have been identified this way (*i.e.*, Ai *et al.* 2015; Kuhlwilm *et al.* 2019; Zhang *et al.* 2019). Finally, as noted by Ottenburghs (2020), recent advances in model-based demographic inference may make it possible to explicitly evaluate ghost introgression scenarios against scenarios involving gene flow between sampled taxa. The vast array of possible ghost introgression scenarios may make model selection difficult, but plausible scenarios can potentially be identified using the approaches described above.

### Distinguishing among models of introgression

Introgression events are often depicted using a phylogenetic network. In these representations, a reticulation edge connects two lineages in the tree that have exchanged genes. However, the placement and orientation of these reticulations can imply specific information about the timing, direction, and species involved in introgression. While methods for inferring introgression are developed under a specific introgression model, many of them are agnostic to the true underlying model when applied to empirical data. More importantly, many methods that infer phylogenetic networks will produce the same network from data generated under very different underlying models (Huson and Bryant 2006). In this section, we highlight the challenges associated with interpreting the results of introgression tests in the context of the underlying model of introgression.

**Figure 6:**
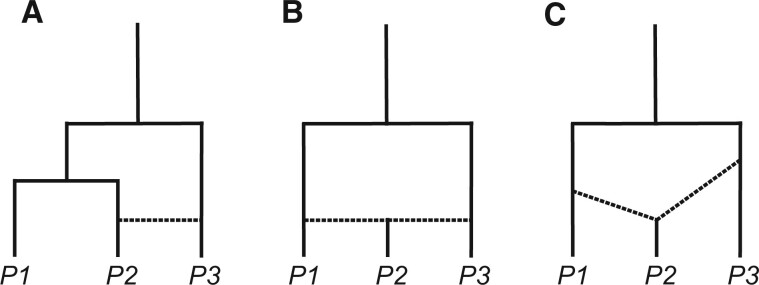
Conceptualizing different models of introgression. (A) Introgression between extant lineages. (B, C) Introgression that results in the formation of a new lineage, differing only with respect to whether there appears to be a period of independent evolution before lineage formation.

Two important models to consider are introgression that occurs between already-existing lineages and introgression that results in the formation of a new lineage. Figure  6A depicts the former scenario, which corresponds to the introgression scenarios considered in the paper thus far. In such cases, a single horizontal reticulation edge is typically used to connect the two taxa involved. This does not naturally convey any information about the direction of introgression, unless the donor and recipient lineages are explicitly identified (*e.g.*, with an arrowhead). In contrast, methods that assume the formation of an admixed population (*e.g.*, Bertorelle and Excoffier 1998; Wang 2003) or hybrid species (*e.g.*, Meng and Kubatko 2009) often use the visualization shown in Figure  6B, where reticulations connect each parent lineage to the newly formed lineage. This could be used to represent, for example, the formation of a new hybrid species (*e.g.*, Rieseberg *et al.* 1995). This representation implies a directionality of introgression without any additional labeling: from the two parent lineages into the newly formed lineage. In both cases, a horizontal reticulation edge can be used to denote the instantaneous exchange of alleles between the involved lineages.

Alternatively, Figure  6C shows an example using nonhorizontal branches, which may imply a period of branching off and independent evolution from the parent species before the hybrid lineage is formed [*e.g.*, Patterson *et al.* (2012), Yu *et al.* (2014), Zhang *et al.* (2018); see Kearns *et al.* (2018) for an empirical example in North American ravens]. An alternative interpretation of this representation is that it shows “standard” introgression involving a now extinct species, in which case the extinct lineage was the donor in the introgression scenario. In this case, there really was a period of independent evolution, but it occurred along a lineage that was not sampled. In all three cases, the placement of the reticulation edge conveys information about the timing of introgression and/or lineage formation.

It important to consider how the methods for detecting introgression discussed here relate to the underlying introgression scenarios, and how this may affect our interpretation of results. Many tests for introgression are agnostic to the particulars of the underlying introgression scenario and will therefore be significant under different models. For example, the *D*-statistic can detect introgression between nonsister taxa regardless of the direction of gene flow (Martin *et al.* 2015; Supplementary Figure S3), or whether introgression results in the formation of a new lineage (Kong and Kubatko 2021). Other methods enforce a particular model of introgression, even though it may not reflect the underlying data. For example, *HyDe* (Blischak *et al.* 2018) is less accurate when estimating the admixture proportion if its hybrid speciation assumption is violated (Kong and Kubatko 2021), while other tests explicitly require the labeling of a putative admixed population under a lineage-formation scenario (Peter 2016). Some statistical methods can explicitly distinguish among these scenarios. The *D*_1_ statistic (Hibbins and Hahn 2019) tests whether gene tree branch lengths are more consistent with hybrid speciation (Figure  6B) or postspeciation introgression (Figure  6A). The MSNC implementation in *BPP* (Flouri *et al.* 2020) may also be able to differentiate among a variety of possible introgression scenarios.

One additional obstacle to distinguishing among models of introgression is a consequence of the information required by machine-readable formats for representing phylogenetic networks. In general, methods return inferred phylogenetic networks in the Extended Newick format (Cardona *et al.* 2008), which requires the specification of a bifurcating “parent” node that occurs closer to the root than the “hybrid” node, which has two incoming lineages. While it is possible for the hybrid node in this format to represent a lateral gene transfer event that does not have a parent closer to the root (Cardona *et al.* 2008), this format is often not used to represent introgression (though it could be).

**Figure 7:**
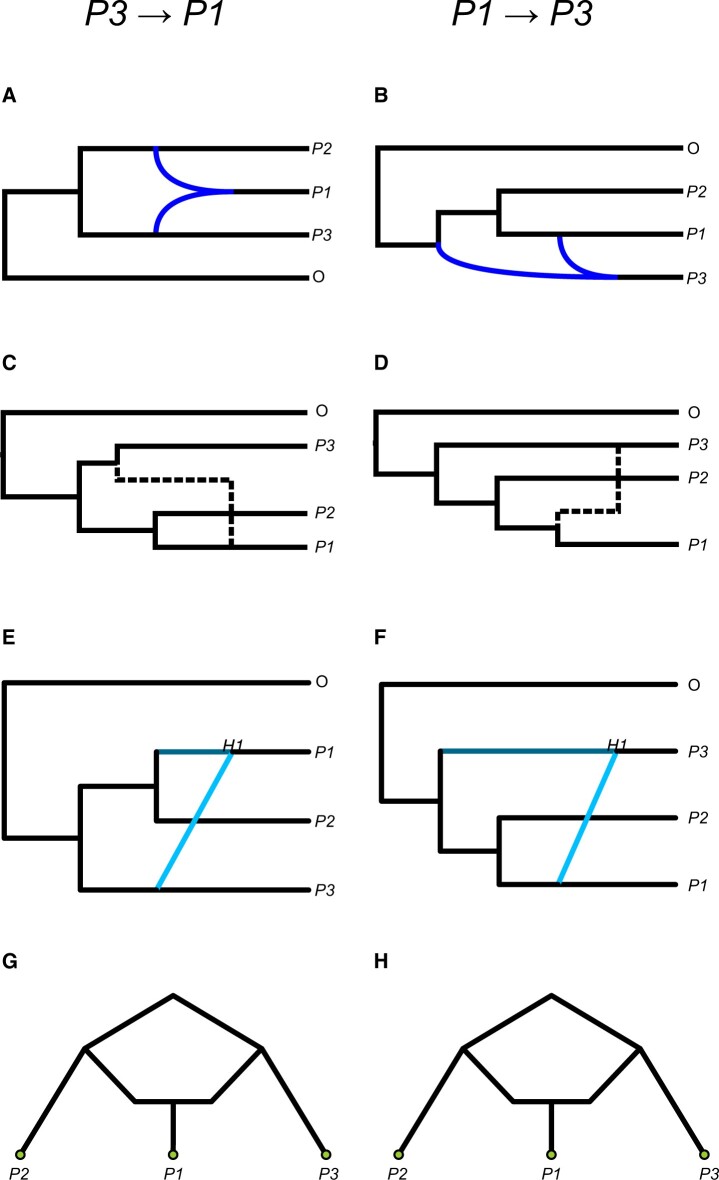
Different visualizations of the same underlying phylogenetic networks. The left column comes from a network representing *P3* → *P1* introgression, while the right column comes from a network representing *P1* → *P3* introgression. The rows, from top to bottom, show visualizations from (A, B) *Dendroscope*; (C, D) *IcyTree*; (E, F) *PhyloPlots*; and (G, H) *admixturegraph*.

Visualizing these results often complicates their interpretation even further. To highlight this, we inferred networks using *PhyloNet*'s *InferNetwork_ML* method (Yu *et al.* 2014) for simulated *P3* → *P1* and *P1* *→* *P3* introgression after speciation (see Supplementary Figure S2) and plotted the results using three popular tools (Figure  7): *Dendroscope* (Huson and Scornavacca 2012), *IcyTree* (Vaughan 2017), and *PhyloPlots*, which is part of the Julia package *PhyloNetworks* (Solís-Lemus *et al.* 2017). All three methods handle the placement of parent and daughter nodes differently. *Dendroscope* visualizes the two incoming lineages to the hybrid node with blue reticulations, which can erroneously imply a lineage-formation or hybrid speciation scenario with *P2* involved in hybridization when introgression is *P3* → *P1* (Figure  7A). Because of the parent/hybrid node structure, all three methods use nonhorizontal reticulations (Figure  7, A–F), which may imply periods of independent evolution in the donor population prior to introgression, even under an instantaneous “pulse” scenario. The general use of reticulations to connect parent and daughter nodes also heavily implies a discrete-time event or series of discrete-time events, rather than a continuous window of gene flow as conceptualized in the IM model. While none of the output networks contained branch lengths, the arbitrary location of placement of the reticulations could imply an inferred time of introgression. We should stress that *PhyloNet’*s *InferNetwork_ML* method was accurate in its inferences about the presence and direction of introgression (Supplementary Figure S3)—it is only the visualization that is misleading.

The visualization of introgression results is especially difficult when information on the timing and direction of gene flow cannot be inferred. The software *admixturegraph* (Leppala *et al.* 2017) plots a network representation solely from the results of a series of *D* tests. We applied this visualization to simulated *P3* → *P1* and *P1* *→* *P3* introgression (Supplementary Figure S2). The resulting plots shown in Figure  7, G and H imply that *P1* formed from hybridization after periods of independent evolution in *P2* and *P3*. However, none of these processes are knowable from a *D*-statistic result (because the direction of introgression cannot be inferred), and this is not the scenario that produced the data. In general, special care should be taken when visualizing the results of *D*-statistics and related test statistics on a phylogeny, since they only provide information on the presence/absence of introgression, and not the direction of introgression.

Clearly differentiating among different possible models of introgression remains challenging. Care should be taken not to overinterpret the results of methods that are model-agnostic, or that rely on a particular model of introgression rather than inferring it from data. This is especially true when interpreting results from common machine-readable visualizations. If possible, hand-drawn “tube tree” representations (*e.g.*, Figure  4) may be more effective in accurately conveying the information available. If automated plotting software is being used, it appears that the visualizations produced by *PhyloPlots* (Figure  7, E and F) are most faithful to the true model of introgression.

### Inferring the number of introgression events

A major challenge that remains in the inference of introgression is how to assess the fit of different numbers of introgression events inferred on the same tree. The mostly widely used methods are formulated to test for the presence of introgression *vs* no introgression but provide no rigorous way to evaluate the number of distinct introgression events. One approach is to perform many quartet-based tests, and then to infer the most parsimonious set of introgression events by collapsing sets of positive tests that share the same ancestral populations (Pease *et al.* 2016; Suvorov *et al.* 2021). However, this approach is highly conservative, as it can collapse cases where there truly are multiple instances of postspeciation introgression within a clade. Additionally, it requires large datasets and the piecing together of many quartets, which makes it impractical in many cases. Nonetheless, such approaches can be used to generate a conservative estimate for the minimum number of introgression events.

Even with likelihood methods, estimating the number of introgression events is not a solved problem. One issue is that adding additional parameters to the likelihood model always improves the likelihood score. This makes it necessary to penalize model complexity when comparing estimated likelihoods. Unfortunately, the information measures that are classically used to perform model selection, such as AIC and BIC, do not adequately scale with the increased complexity of adding a new reticulation to a phylogenetic network. This is because adding a new reticulation does not just add a single new model parameter—it adds a whole new space of possible networks (Blair and Ané, 2020). AIC and BIC penalize the increased complexity of model parameters, but not the increased complexity of models within a set of parameters. The problem is greater for methods based on pseudolikelihood such as *SNaQ*, because these information measures are not intended for pseudolikelihood estimates. Bayesian approaches such as those implemented in *PhyloNet* (Wen and Nakhleh 2018) and *SpeciesNetwork* can incorporate appropriate penalties for model complexity, but unfortunately scale poorly to larger datasets and larger numbers of reticulations (Elworth *et al.* 2019).

While no methods currently exist that can both explicitly penalize model complexity and scale to large datasets, there are several alternate approaches available for assessing the fit of phylogenetic networks. One simple, empirical approach is to use a slope heuristic where networks are inferred across different numbers of reticulations, and the best network is taken as the least complex one after which the likelihood score appears to stop improving. This is the method recommended for use with *SNaQ* (Solís-Lemus and Ané 2016). *PhyloNet* has methods that can evaluate the fit of a network using *k*-fold crossvalidation or parametric bootstrapping (Yu *et al.* 2014), which can both address this problem. Finally, a promising approach from Cai and Ané (2021) involves using the MSNC to calculate the quartet concordance factors expected from an estimated network. A goodness-of-fit function is then used to evaluate the fit of these expected concordance factors to those observed in the data. This is similar to the method implemented in *admixturegraph* (Leppala *et al.* 2017) for use with *D-*statistics.

## Conclusions 

In conclusion, several methodological and technical challenges remain in the inference of introgression, including: more accurate estimation of the extent, timing, and direction of introgression; detection of introgression between sister taxa; spurious results generated by unsampled lineages; inference of the number of introgression events in a clade; and accurate automated visualization of phylogenetic networks. Despite these challenges, currently available approaches have remarkable power to detect and characterize introgression under a wide variety of conditions, especially when used in a complementary fashion. Overall, these methods will continue to reveal the nature and influence of introgression throughout the natural world.

## Data availability

Parameters used to generate our simulated data are summarized in Supplementary Figure S2 and Table S1. Scripts and processed data related to the simulation study are available at https://github.com/mhibbins/introgression_review (last accessed October 18, 2021).

Supplementary material is available at *GENETICS* online.

## Supplementary Material

iyab173_Supplementary_Data
