# Supplementary material for: Phylogenomic approaches to detecting and characterizing introgression
Source: Genetics. 2021 Nov 11;220(2):iyab173. doi: 10.1093/genetics/iyab173 (PMC9208645; doi:10.1093/genetics/iyab173)
Supplement: iyab173_Supplementary_Data [file iyab173_supplementary_data.pdf]

Supplementary Materials and Methods for Hibbins & Hahn 2021

Simulation study under different introgression scenarios

To illuminate many of the patterns and approaches discussed in this review, we conducted a small simulation study. We used the five introgression scenarios shown in Figure 2, as well as one scenario with only ILS and several additional scenarios involving ghost introgression (Supplementary Figure 2). Introgression was simulated in *ms* by specifying an instantaneous population split and join event; this is equivalent to simulating under the multispecies network coalescent framework (Hibbins and Hahn 2019). For each set of conditions, we performed 100 replicate simulations each consisting of 3000 gene trees with branch lengths. We evaluated the performance of three different test statistics designed to capture slightly different information about introgression: D, D_3, and Δ. In addition, we applied the InferNetwork_ML method (Yu et al. 2014) in *PhyloNet*, which infers a phylogenetic network using maximum-likelihood. For the three test statistics, we evaluated significance by bootstrap-resampling the gene trees in each dataset to estimate the sampling variance. The *z*-score obtained from bootstrap-resampling was used to estimate a two-tailed *p*-value. The method we use in *PhyloNet* evaluates the fit of a phylogenetic network internally (Yu et al. 2012) using a combination of the model selection measures AIC (Akaike 1974), AICc (Burnham and Anderson 2002), and BIC (Schwarz 1978). For our purposes, a positive result was taken as any result where *PhyloNet* selected a network over a strictly bifurcating tree. See Supplementary Table 1 for the simulation parameters used for each condition.

The power of each method to detect introgression under each scenario is shown in Supplementary Figure 3. All four methods yielded low false positive rates in the presence of high ILS but no introgression, confirming that they are effective tests against an ILS-only null hypothesis. For non-sister taxa, *PhyloNet* was always capable of identifying introgression, while the power of the other methods was strongly affected by the direction of introgression. A reduction of power for *P1* → *P3* introgression is consistent with the effect of direction on gene tree branch lengths described above, but the magnitude of the reduction is somewhat surprising: there is almost three times as much power to detect *P3* → *P1* introgression. Of the four methods, only *PhyloNet* appears capable of reliably inferring introgression between sister lineages, again consistent with expectations.

The D and Δ statistics, as well as *PhyloNet*, did not give significant results when introgression occurred between *P1* and an unsampled ingroup lineage. The D_3 statistic, interestingly, does appear to be sensitive to this scenario, at least when the ghost population is the donor. This suggests that patterns of pairwise divergence may be especially useful for detecting introgression with unsampled populations. When introgression occurs between *P1* and an outgroup ghost lineage, there is no effect when the ghost is the recipient, while all four methods are strongly affected when the ghost is the donor. These observations are consistent with expectations for ghost populations, highlighting the importance of careful interpretation of the potential taxa involved in a positive result. In this case, all methods appear to suggest introgression between *P2* and *P3*, even though neither of these lineages was involved in the introgression. This occurs

because introgression from outside the rooted triple draws *P1* to the outside as well, leaving *P3* more closely related to *P2*.

In addition to testing for the presence of introgression, we evaluated the ability of *PhyloNet* to infer the direction of introgression, and of several methods to infer the rate of introgression. We evaluated the ability of *PhyloNet* to correctly identify the taxa involved, the donor and recipient lineages, and the rate of introgression. For the two conditions involving introgression between non-sister taxa, we additionally estimated the rate of introgression using the D_p statistic and an analogous version of the Δ statistic where the count of the concordant tree topology was added to the denominator; we refer to this statistic as Δ_p.

We found that *PhyloNet* was highly accurate at identifying the taxa and direction for *P1* → *P3* introgression (Supplementary Figure 3). However, somewhat surprisingly, it often failed to identify the taxa involved when introgression was *P3* → *P1* (although it always correctly identified that introgression had occurred somewhere). While it is more difficult to detect introgression in the *P1* → *P3* direction, once it is detected it appears that the additional signal in gene tree branch lengths makes it easier for *PhyloNet* to infer the direction. For sister lineages, *PhyloNet* always correctly identified the taxa, but cannot accurately infer the direction. However, *PhyloNet* must always specify the direction of introgression, and its behavior differs between scenarios. For introgression between extant sister species, the direction of introgression appears to be assigned randomly, while for ancestral sister species introgression is always inferred to be in one direction. For the rate of introgression, *PhyloNet* appears to slightly overestimate the true rate under all scenarios in which it correctly identified introgression (Supplementary Figure 4). By contrast, D_p and Δ_p tend to slightly underestimate the rate of introgression between non-sister taxa (Supplementary Figure 4).

Supplementary Figures and Tables

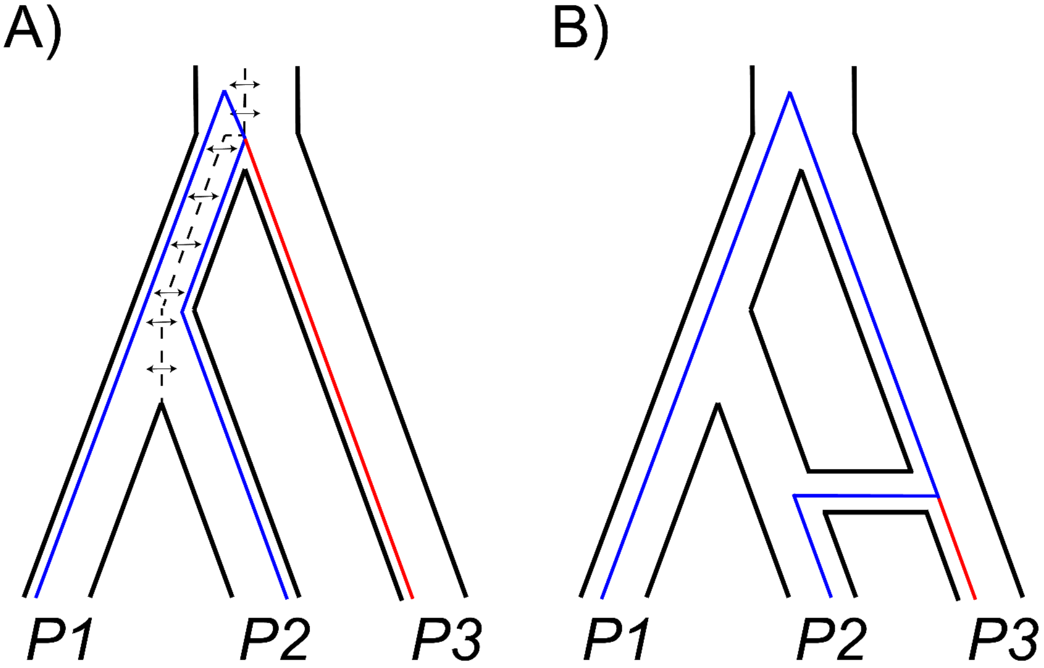

Supplementary Figure 1: Distinguishing ancestral population structure (A) from introgression (B). Persistent structure in the ancestral population of a quartet, which may or may not continue after the first speciation event, can result in the same asymmetries in gene tree topologies and divergence times that are expected from introgression between non-sister taxa. These two scenarios are distinguishable by studying the distribution of branch lengths, in particular the length of the tip branch leading to *P3* (red).

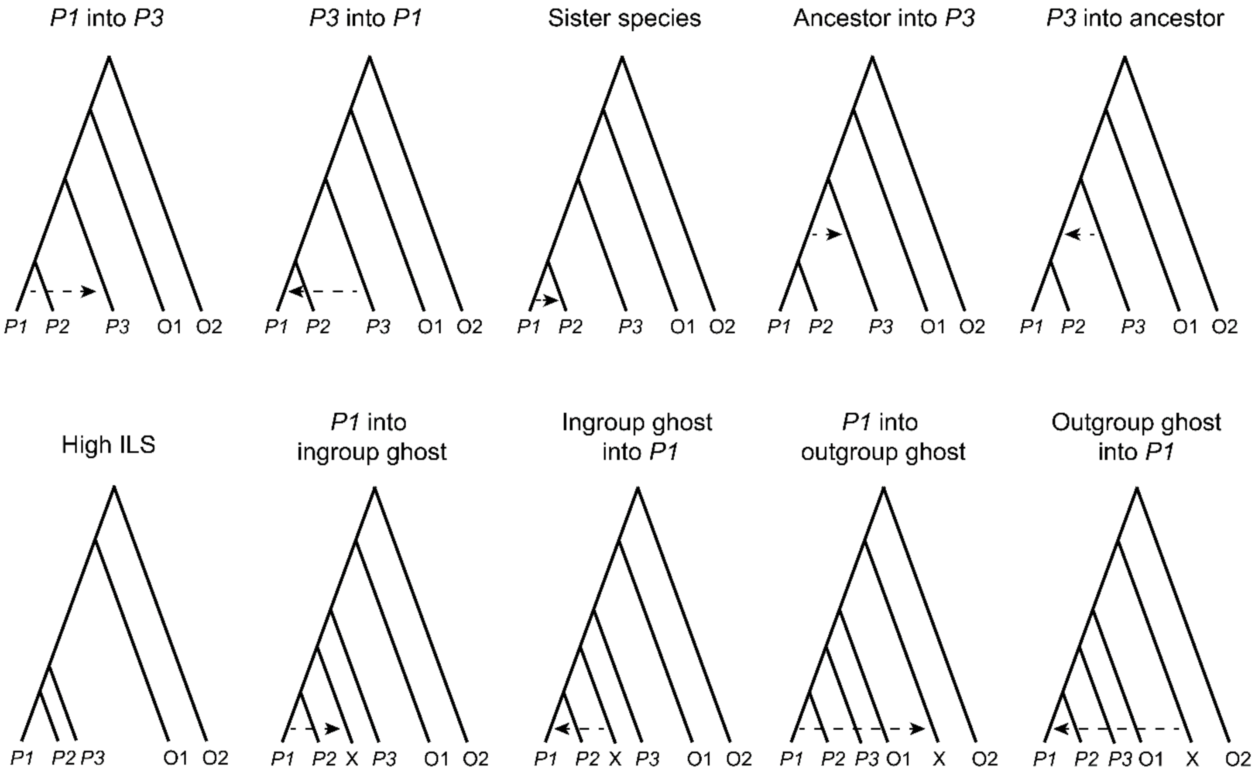

Supplementary Figure 2: A visual overview of the ten different conditions used in our simulation study. Branch lengths are not to scale.

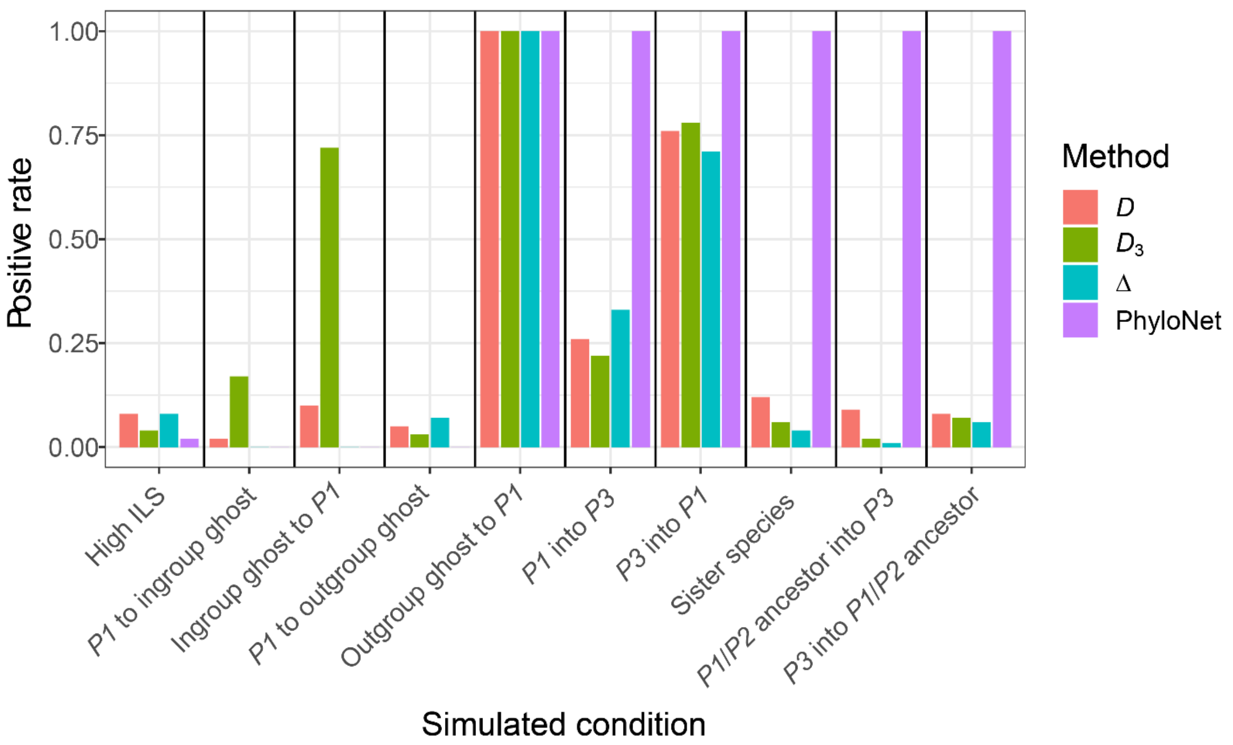

Supplementary Figure 3: Power (y-axis) of four different methods (color legend) to infer the presence of introgression across ten different simulation conditions (x-axis). Power is measured as the proportion of tests that are significant; for the "High ILS" condition it therefore represents the false positive rate.

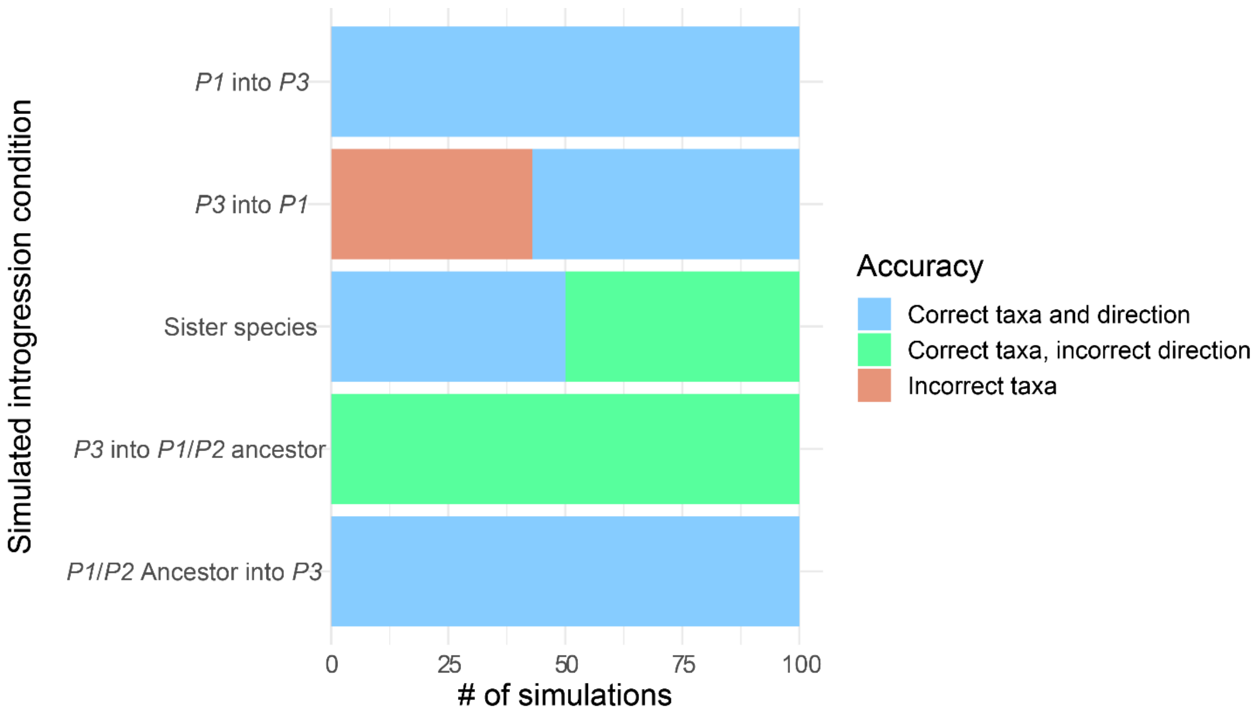

Supplementary Figure 4: The power of *PhyloNet* to identify the taxa involved and direction of introgression across five simulation conditions.

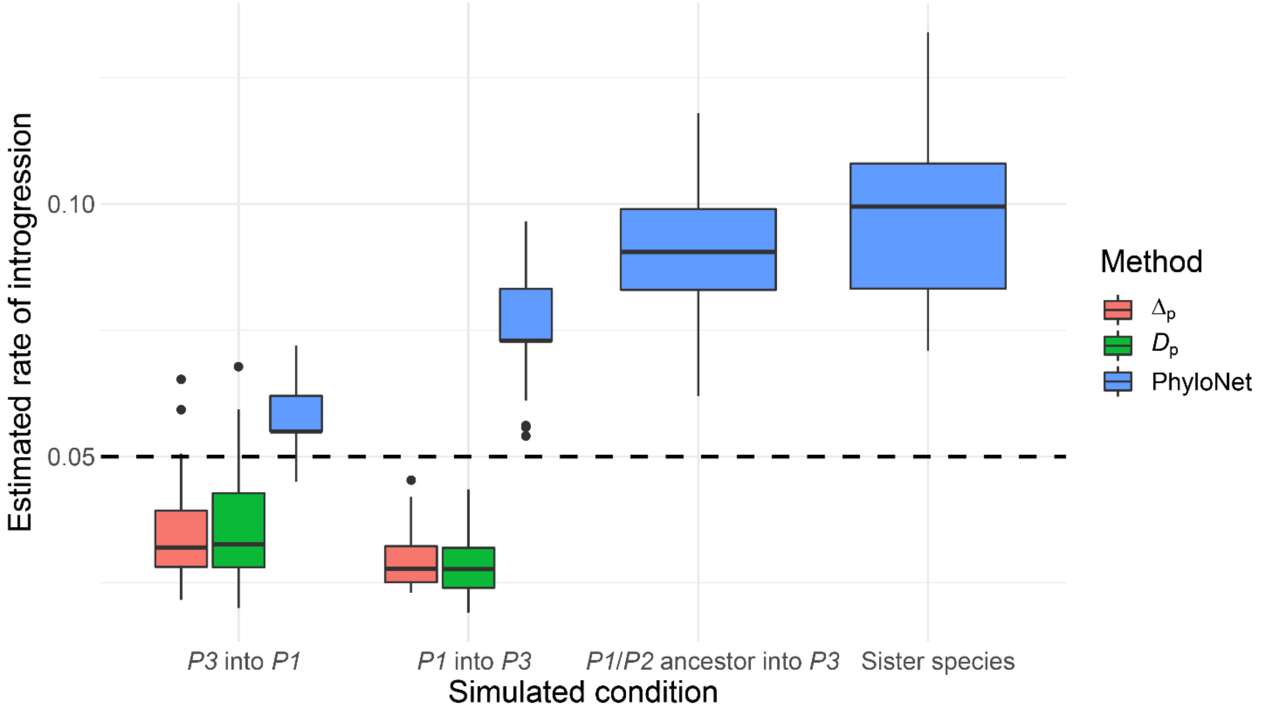

Supplementary Figure 5: Accuracy of three methods (color legend) for estimating the rate of introgression (y-axis) across four simulation conditions (x-axis). The horizontal dashed line shows the true simulated rate of introgression.

Condition	P1/P2_split	P1P2/P3_split	P1P2P3/O1_split	O1/O2_split	intro_timing	intro_rate	ghostpop_split	theta
P1 into P3	0.6	1.2	8	20	0.3	0.05	N/A	0.005
P3 into P1	0.6	1.2	8	20	0.3	0.05	N/A	0.005
Sister species	0.6	1.2	8	20	0.3	0.05	N/A	0.005
Ancestor into P3	0.6	1.2	8	20	0.9	0.05	N/A	0.005
P3 into ancestor	0.6	1.2	8	20	0.9	0.05	N/A	0.005
High ILS	0.6	0.62	8	20	N/A	0.05	N/A	0.005
P1 into ingroup ghost	0.6	8	20	30	0.3	0.05	1.2	0.005
Ingroup ghost into P1	0.6	8	20	30	0.3	0.05	1.2	0.005
P1 into outgroup ghost	0.6	1.2	8	30	0.3	0.05	20	0.005
Outgroup ghost into P1	0.6	1.2	8	30	0.3	0.05	20	0.005

Supplementary Table 1: Parameters used for introgression simulation conditions in *ms*. Split times and theta are in units of *2N* generations.
